# Effect of dietary resveratrol supplementation on growth performance, antioxidant capacity, intestinal immunity and gut microbiota in yellow-feathered broilers challenged with lipopolysaccharide

**DOI:** 10.3389/fmicb.2022.977087

**Published:** 2022-08-25

**Authors:** Zhentao He, Yaojie Li, Taidi Xiong, Xiaoyan Nie, Huihua Zhang, Cui Zhu

**Affiliations:** School of Life Science and Engineering, Foshan University, Foshan, China

**Keywords:** lipopolysaccharide, resveratrol, yellow-feathered broilers, growth performance, gut microbiota

## Abstract

Resveratrol (RES) displays strong antioxidant and anti-inflammatory properties in protecting the animals from various stressors and inflammatory injuries, but its interrelationship with the gut microbiota remained largely unclear. This study was carried out to investigate the effects of dietary RES supplementation on growth performance, antioxidant capacity, intestinal immunity and gut microbiota in yellow-feathered broilers challenged by lipopolysaccharide (LPS). A total of 240 yellow-feathered broilers were randomly assigned to four treatment groups in a 2 × 2 factorial design. The broilers were fed with the control diet or control diet supplemented with 400 mg/kg RES, followed by challenge with LPS or the same amount of saline. Dietary RES supplementation significantly alleviated the decreases in the final body weight (BW), average daily gain (ADG), and ADFI induced by LPS (*P* < 0.05). LPS challenge significantly increased plasma concentrations of triglyceride, high-density lipoprotein cholesterol (HDL-C), aspartate aminotransferase (AST), and cortisol levels, but decreased triiodothyronine (T3) and insulin levels (*P* < 0.05). Dietary supplementation with RES significantly reversed the elevated creatinine concentrations and the decreased concentrations of T3 and insulin caused by LPS (*P* < 0.05). Moreover, dietary RES supplementation significantly increased plasma total antioxidant capacity (T-AOC) and catalase (CAT) activities and superoxide dismutase (SOD) and T-AOC activities in jejunal mucosa and reduced malondialdehyde (MDA) concentration in the plasma (*P* < 0.05). The reduction in the villus height to crypt depth ratio in duodenum, jejunum and ileum and the shortening of villus height in jejunum and ileum caused by LPS were also alleviated by RES treatment (*P* < 0.05). Furthermore, the increased concentrations of intestinal tumor necrosis factor-α (TNF-α), interleukin-6 (IL-6), and IL-1β caused by LPS were significantly decreased by RES treatment (*P* < 0.05). Dietary RES treatment increased the mRNA expression of *claudin-1*, *claudin-5*, *occludin*, and *zonula occludens-1* (*ZO-1*), and decreased mRNA expression of *IL-1*β, *IL-8*, *IL-17*, and *TNF-*α after LPS challenge (*P* < 0.05). Dietary RES treatments significantly decreased the dominance of cecal microbiota, and increased the Pieiou-e and Simpson index. Moreover, dietary RES supplementation increased relative abundance of *UCG_ 009*, *Erysipelotrichaceae*, *Christensenellaceae_R-7_group*, *Anaerotruncus*, *RF39*, and *Ruminococcus* while decreasing the abundance of *Alistipes* at genus level. Spearman correlation analysis revealed that the microbes at the order and genus levels significantly correlated with indicators of growth performance, antioxidant capacity, and intestinal health. Collectively, dietary supplementation with 400 mg/kg RES could improve growth performance and antioxidant capacity, and modulate intestinal immunity in yellow-feathered broilers challenged by LPS at early stage, which might be closely associated with the regulation of gut microbiota community composition.

## Introduction

With the increasing demands for animal products, the intensive systems has greatly developed over the last few decades. However, the improvement of intensive production makes poultry more vulnerable to various stressors including pathogenic and toxin infections (Kumar et al., 2012). Immune stress represents a great threat to the poultry farming industry. Under immune stress, the overproduction of inflammatory cytokines would lead to impaired intestinal structure and barrier function, thereby affecting the nutrient digestion and absorption and increasing the risk of intestinal inflammation and diseases (Chen et al., 2018; Sun et al., 2021). Moreover, prolonged immune stress can cause suppression of immune function, especially in the early growth stage when the immune system of broiler has not yet well developed (Song et al., 2021). Lipopolysaccharide (LPS) is a major component of the outer membrane of Gram-negative bacteria and has been widely used as a non-pathogenic immune activator to induce immune stress in broilers. Therefore, finding the effective nutrient regulation strategies to protect the intestinal health and maintain immune function are important for improving the production efficiency and disease resistance of poultry.

Resveratrol (RES) (*trans*-3,5,4′-trihydroxystilbene) is a naturally polyphenol found in a variety of plants, and displays strong antioxidant and anti-inflammatory properties (Liang et al., 2019; Meng et al., 2021), which has great application prospects in animal production. Previous study has shown that RES can effectively alleviate the damages of intestinal morphology and barrier integrity induced by heat stress in broilers (He et al., 2019a,^2020^). Moreover, dietary RES treatment can reduce diarrhea and intestinal inflammation in piglets by altering intestinal gene expression and gut microbiota (Meng et al., 2019). Indeed, gut microbiota play an important role in the digestion and absorption of nutrients and in the immune response of animals, thus affecting the growth development and health of the host animal (Yao et al., 2021). Previous study has shown that the improvement of growth performance and immunity might be associated with the balanced cecum microbiota in broilers challenged by *Escherichia coli* (Mohebodini et al., 2019). However, it remained unclear whether the protective effect of RES on the growth performance and intestinal health was involved with the changes of gut microbiota composition and diversity in yellow-feathered broilers challenged with LPS.

Therefore, the purpose of this experiment was to investigate the effects of dietary supplementation with RES on growth performance, plasma biochemical parameters, hormones levels, antioxidant capacity and intestinal health, and tempted to identify their potential relationships with the alterations of gut microbiota in yellow-feathered broilers challenged with LPS.

## Materials and methods

### Experimental design, diets and management

The experimental protocol (FOSU2022004) was approved by Animal Care and Use Committee of Foshan University (Foshan, China). A total of 240 healthy 1-day-old yellow-feathered broiler male chicks with initial body weight (BW) of 34.43 ± 0.24 g were randomly divided into four treatment groups in a 2 × 2 factorial design, which included the control group (CON), RES group, LPS group, and RES + LPS group. The broilers in CON group and LPS group were fed the basal diet, while those in RES group or RES + LPS group were fed with the basal diet supplemented with 400 mg/kg RES. Each treatment group had five replicates of 12 birds each replicate. The basal diet (Table 1) was formulated according to the nutritional levels recommended by Ministry of Agriculture of the People’s Republic of China. (2020). RES (purity ≥ 98%) was purchased from Shaanxi Sciphar Natural Products Co., Ltd., while LPS (*E. coli* O55:B5) was provided from Sigma-Aldrich (Saint Louis, MO, United States).

**TABLE 1:** The composition and nutrient levels of the basal diet for yellow-feathered broilers.

Ingredients	%	Calculated nutrient levels^1^	%
Corn	39.07	MJ (kcal/kg)	12.35
Wheat	25.00	CP	21.50
Soybean meal (43% CP)	22.00	EE	3.40
Peanut meal	3.00	CF	2.50
Corn gluten meal (58% CP)	5.00	Ca	0.90
Limestone	1.45	Total P	0.60
CaHPO_4_	1.10	L-Lysine	1.38
L-lysine sulfate	0.64	DL-Met	0.61
DL-Met	0.32	L-Thr	0.82
NaCl	0.28		
L-Thr	0.19		
Lard oil	1.45		
Vitamin-mineral premix^2^	0.50		
Total	100.00		

^1^Values were calculated from data provided by China Nutrient Requirements for yellow-feathered broilers (2020).

^2^The premix provided the following per kg of diet: VA, 6,000 IU; VD_3_, 2,000 IU; VE, 30 mg; VK_3_, 2 mg; VB_1_, 3 mg; VB_2_, 5 mg; pantothenic acid, 800 mg; choline chloride 1,500 mg; nicotinic acid, 30 mg; pyridoxine, 3 mg; folic acid, 500 mg; biotin, 0.2 mg; VB_12_, 1 mg; Fe, 100 mg; Cu, 8 mg; Mn, 100 mg; Zn, 100 mg; I, 0.42 mg; Se, 0.3 mg.

The experiment lasted 21 days. The chickens of each replicate were kept in individual cage and had free access to feed and drinking water during the whole experiment. At days 16, 18, and 20 of the experiment, the broilers in LPS group and RES + LPS group were injected intraperitoneally with LPS solution (1 mg/kg BW, LPS dissolved in 0.9% saline at a concentration of 1 mg/mL), while the broilers in CON and RES groups were received the same amount of sterilized saline. The challenge dosage and treatment of LPS was chosen accordingly to the previous study (Han et al., 2020).

### Sample collection

The boilers in each group were weighed on days 1, 16, 18, 20, and 21 of the experiment, and one chicken with average BW of each replicate was selected for sample collections on day 21. After fasting for 12 h, the blood (10 mL) was collected from the wing veins of the chickens, and centrifuged at 3,000 rpm for 10 min to get the plasma samples for the determination of plasma biochemical parameters, hormone levels and antioxidant indicators. After blood collection, the chickens were subjected for slaughter. Briefly, the middle sections (about 2 cm) of the duodenum, jejunum, and ileum were collected and placed in 10% formalin fixation to determine the intestinal morphology. Then, the mucosal samples were gently scraped from another portion of middle jejunum (about 10 cm) after rinsing with ice-cold saline, and placed into 1.5 mL EP tubes for the determinations of antioxidant indicators and intestinal cytokines. Moreover, the samples of cecum contents were collected and immediately snap-frozen in liquid nitrogen and stored at −80°C to determine the changes of gut microbiota in yellow-feathered broilers.

### Determinations

#### Growth performance

Feed consumption of each replicate was recorded before each weighing or in case of mortality. The average daily gain (ADG), average daily feed intake (ADFI), and feed-to-gain ratio (F/G) were calculated accordingly.

#### Plasma biochemical parameters and hormones concentrations

The plasma biochemical parameters, including total protein, uric acid, glucose, cholesterol, triglyceride, high-density lipoprotein cholesterol (HDL-C), low-density lipoprotein cholesterol (LDL-C), none-esterified fatty acid (NEFA), total bile acid, aspartate aminotransferase (AST), alanine aminotransferase (ALT), creatine kinase, creatine kinase-MB, and creatinine concentrations were determined according to the methods described previously (Zhu et al., 2020).

The plasma concentrations of hormones including triiodothyronine (T3), thyroxine (T4), insulin, and cortisol concentrations were determined by commercial Elisa kits according to the instructions of the manufacturer (Beijing North Institute of Biotechnology Co., Ltd., Beijing, China).

#### Analyses of antioxidant capacity in the plasma and jejunal mucosa

The antioxidant indexes of plasma and jejunal mucosa samples including glutathione peroxidase (GSH-Px), superoxide dismutase (SOD), catalase (CAT), total antioxidant capacity (T-AOC), and malondialdehyde (MDA) were measured using the commercial kits from Nanjing Jiancheng Institute of Biological Engineering (Nanjing, China) according to the manufacturer’s instructions.

#### Measurement of intestinal morphology

The mid-sections of duodenum, jejunum, and ileum were fixed in 10% formalin for 24 h. The fixed samples were embedded in paraffin, and cross sections of each sample were fixed on slides coated with polylysine, deparaffinized, rehydrated, and then stained with hematoxylin-eosin (HE). Sections were observed using an Olympus MD50-T microscope. The villus height and crypt depth of at least 25 villus samples were measured by MShot Image Analysis System (Guangzhou, China), and the ratio of villus height to crypt depth was calculated accordingly.

#### Measurement of intestinal cytokine secretions

Intestinal cytokines including intestinal tumor necrosis factor-α (TNF-α), interleukin-6 (IL-6), IL-1β, IL-17, and transforming growth factor-β (TGF-β) were quantified using the commercial Elisa kits according to manufacturer protocols (Cusabio, Barksdale, DE, United States).

#### RNA extraction, cDNA synthesis and real time quantitative PCR

Total RNA was isolated from jejunal mucosa samples using TRIzol reagent (Invitrogen, Carlsbad, CA, United States) according to the manufacturer’s instructions. The purity and concentration of RNA were determined using a model DS-11 spectrophotometer (DeNovix, Wilmington, DE, United States). The total RNA (1 μg) was used to synthesize cDNA using a T100 Thermal Cycler (Bio-Rad, Hercules, CA, United States). The real time quantitative PCR (RT-qPCR) analysis was performed in triplicate using a final volume of 10 μL of SYBR Green (Bio-Rad, Hercules, CA, United States) by a 10-fold dilution of cDNA. The targeted gene-specific primers (Table 2) were designed by Primer 5.0 and synthesized by Tsingke Biotechnology Co., Ltd. (Beijing, China). The RT-qPCR conditions were 95°C for 5 min, followed by 40 amplification cycles (95°C for 15 s, 60°C for 30 s, and 72°C for 30 s). The β-actin was used as an internal control and the fold change of targeted genes was calculated with β-actin for each sample using the 2^–ΔΔ*Ct*^ method.

**TABLE 2:** Sequence, product size and accession number of primers for quantitative real-time PCR.

Item	Nucleotide sequence of primers (5′→3′)	Product size (bp)	GenBank accession
*Claudin-1*	F: CATACTCCTGGGTCTGGTTGGT	100	NM 001013611.2
	R: GACAGCCATCCGCATCTTCT		
*Claudin-5*	F: CATCACTTCTCCTTCGTCAGC	111	NM_204201
	R: GCACAAAGATCTCCCAGGTC		
*Occludin*	F: ACGGCAGCACCTACCTCAA	123	XM 025144247.2
	R: GGGCGAAGAAGCAGATGAG		
*ZO-1*	F: CTTCAGGTGTTTCTCTTCCTCCTC	131	XM 030016322.2
	R: CTGTGGTTTCATGGCTGGATC		
*IL-1*β	F: ACTGGGCATCAAGGGCTACA	142	Y15006.1
	R: GCTGTCCAGGCGGTAGAAGA		
*IL-6*	F: CTCCTCGCCAATCTGAAGTC	99	NM_204628
	R: CCTCACGGTCTTCTCCATAAAC		
*IL-8*	F: GGCTTGCTAGGGGAAATGA	200	DQ393272.2
	R: AGCTGACTCTGACTAGGAAACTGT		
*IL-17*	F: CTCCTCTGTTCAGACCACTGC	126	AJ493595.1
	R: ATCCAGCATCTGCTTTCTTGA		
*TGF-*β	F: AGGATCTGCAGTGGAAGTGGAT	138	M31160
	R: CCCCGGGTTGTGTTGGT		
*TNF-*α	F: GAGCGTTGACTTGGCTGTC	64	GU230788.1
	R: AAGCAACAACCAGCTATGCAC		
β*-actin*	F: ATGATATTGCTGCGCTCGTT	145	L08165.1
	R: TCTTTCTGGCCCATACCAACC		

ZO-1, zonula occludens-1; IL-1β, interleukin-1β; IL-6, interleukin-6; IL-8, interleukin-8; IL-17, interleukin-17; TGF-β, transforming growth factor-β; TNF-α, tumor necrosis factor-α.

#### Analysis of gut microbial composition and diversity

The 16S RNA sequencing technique was used to determine the potential changes in the composition and diversity of the gut microbiota in yellow-feathered broilers fed with RES-supplemented diets followed by challenge with LPS. Microbial DNA was extracted from cecum contents using a DNA kit (Omega Bio-tek, Norcross, GA, United States) according to the manufacturer’s instructions. The qualified extracted DNA samples were then diluted to 1 ng/μL using sterile water and the V3–V4 variable region of the 16S rRNA gene was amplified using specific primers (341 F: 5′-CCTAYGGGRBGCASCAG-3′; 806 R: 5′-GGACTACNNGGGTATCTAAT-3′). PCR amplicons were purified using the Qiagen Gel Extraction Kit (Qiagen, Hilden, Germany) according to the manufacturer’s instructions. The PCR products that meet the library construction requirements were used for library construction using TruSeq DNA PCR-Free Sample preparation library construction kit (Illumina, San Diego, CA, United States) and then sequenced by HiSeq 2500 PE 250 platform (Novogene Bioinformatics Technology Co., Ltd., Tianjin, China). The 16S rRNA sequencing data was performed using the QIIME software package. The high-quality sequences of operational taxonomic units (OTUs) at a 97% identity were aligned against the SILVA database (Ribocon GmbH, Bremen, Germany) after removing chimeric sequences *via* UCHIME software (Tiburon, CA, United States). The Venn diagram with shared and unique OTUs was used to identify the similarity and difference among treatments. The alpha-diversity parameters included Chao 1, Dominance Observed-OTUs, Pieiou-e, Shannon index, and Simpson index. The principal coordinate analysis (PCoA) and non-metric multidimensional scaling (NMDS) were accessed to calculate the β-diversity between groups. The differences in the relative abundances of microbiota among treatments were compared using the linear discriminant analysis effect size (LEfSe) and Metastat analysis. The Spearman correlations between the representative bacteria at phylum, order, and genus levels and the significant phenotype parameters concerning growth performance, antioxidant capacity, intestinal health were plotted as heatmaps using the R package (version 2.15.3).

### Statistical analysis

The data were subjected to two-way ANOVA by SPSS 23.0 (SPSS, Inc., Chicago, IL, United States) to analyze the main effects of LPS or RES and their interactions. Significant differences between means were compared using Duncan’s multiple comparison. The replicates (*n* = 5) were considered as experimental units. The results were expressed as mean and combined standard error (SEM). *P* < 0.05 was considered as significantly different while *P* < 0.10 indicated a trend.

## Results

### Growth performance

As shown in Table 3, LPS challenge caused significant decreases in final BW at days 21, ADG and ADFI during days 16–21 (post-challenge with LPS) and the whole period (days 1–21) in yellow-feathered broilers (*P* < 0.01), but dietary supplementation with 400 mg/kg RES significantly alleviated the decreases in these growth performance parameters during this period caused by LPS (*P* < 0.05). Moreover, no significant RES × LPS interaction for final BW, ADG, ADFI, or F/G during days 16–21 was observed after LPS challenge (*P* > 0.05). Additionally, dietary supplementation with 400 mg/kg RES significantly increased the ADFI during days 1–16 (pre-challenge) in yellow-feathered broilers compared to those fed with the basal diets (*P* < 0.05) (data not shown).

**TABLE 3:** Effect of dietary resveratrol supplementation on growth performance in yellow-feathered broilers with lipopolysaccharide^1^.

Item	Treatment				SEM	*P*-value		
	CON	RES	LPS	RES + LPS		LPS	RES	LPS × RES
BW 16 days (g)	353.97	360.46	353.59	354.13	7.38	0.529	0.510	0.575
BW 21 days (g)	468.12^b^	485.77^a^	447.19^c^	466.67^b^	6.75	0.001*	0.001*	0.847
**Day 16–21**								
ADG (g/d)	19.03^b^	20.88^a^	15.59^c^	18.76^b^	0.54	<0.001*	<0.001*	0.108
ADFI (g/d)	47.17^b^	48.39^a^	45.34^c^	47.70^ab^	0.41	0.001*	<0.001*	0.067
F/G	2.48^bc^	2.32^c^	2.92^a^	2.54^b^	0.09	<0.001*	0.001*	0.110
**Day 1–21**								
ADG (g/d)	19.62^b^	20.43^a^	18.71^c^	19.68^b^	0.32	0.002*	0.001*	0.738
ADFI (g/d)	33.34^bc^	34.32^a^	32.70^c^	33.62^b^	0.32	0.008*	0.001*	0.878
F/G	1.70	1.68	1.75	1.71	0.03	0.061	0.111	0.631

^1^Values are means and standard error of the means (*n* = 5).

BW, body weight; ADG, average daily gain; ADFI, average daily feed intake; F/G, feed to gain ratio; CON, control; RES, resveratrol; LPS, lipopolysaccharide; RES + LPS, dietary resveratrol treatment followed by LPS challenge.

^*abc*^Means in the same row with different superscripts differ (*P* < 0.05).

The * indicates statistically significant difference (*P* < 0.05).

### Plasma biochemical parameters and hormones concentrations

Lipopolysaccharide challenge resulted in significant increase of plasma concentrations of triglyceride, HDL-C, AST, total bile acid, creatine kinase, creatine kinase-MB and creatinine in yellow-feathered broilers (*P* < 0.05) (Table 4). In contrast, dietary supplementation with 400 mg/kg RES significantly alleviated the elevated creatinine concentrations and decreased the concentration of creatine kinase-MB caused by LPS (*P* < 0.05). Moreover, there was a significant RES × LPS interaction in plasma NEFA concentrations of yellow-feathered broilers (*P* = 0.038). However, the plasma concentrations of total protein, glucose, urea acid, cholesterol, ALT, and LDL-C in yellow-feathered broilers were not significantly changed among different treatments (*P* > 0.05).

**TABLE 4:** Effect of dietary resveratrol supplementation on plasma biochemical parameters and hormones concentrations in yellow-feathered broilers with lipopolysaccharide^1^.

Item	Treatment				SEM	*P*-value		
	CON	RES	LPS	RES + LPS		LPS	RES	LPS × RES
Total protein (g/L)	31.83	32.53	29.17	32.13	1.98	0.305	0.226	0.441
Urea acid (μmol/L)	156.33	139.67	215.33	286.00	34.40	0.062	0.372	0.801
Glucose (mmol/L)	13.10	13.17	12.06	12.27	1.12	0.258	0.868	0.935
Triglyceride (mmol/L)	0.35^b^	0.34^b^	0.44^a^	0.40^ab^	0.03	0.003*	0.246	0.480
Cholesterol (mmol/L)	4.22	3.92	4.63	4.22	0.33	0.072	0.382	0.738
HDL-C (mmol/L)	2.70^b^	2.66^b^	3.25^a^	2.83^b^	0.16	0.015*	0.076	0.147
LDL-C (mmol/L)	0.86	0.73	0.91	0.91	0.13	0.240	0.493	0.472
NEFA (mmol/L)	0.76	0.96	1.01	0.77	0.14	0.777	0.808	0.038*
AST (U/L)	171.0^ab^	162.2^b^	185.2^a^	177.2^ab^	7.15	0.011*	0.116	0.938
ALT (U/L)	1.25	1.00	1.50	1.25	0.32	0.295	0.295	1.000
Total bile acids (umol/L)	4.16^b^	3.64^b^	7.28^a^	5.74^ab^	1.60	0.015*	0.301	0.604
Creatine kinase (U/L)	1073.33^ab^	906.33^b^	1299.67^a^	1130.67^ab^	114.96	0.024*	0.073	0.990
Creatine kinase-MB (U/L)	1285.27^a^	962.30^b^	1426.00^a^	1321.33^a^	70.81	0.001*	0.003*	0.061
Creatinine (μmol/L)	11.20^b^	9.00^b^	17.20^a^	11.60^b^	2.57	0.031*	0.048*	0.364
T3 (ng/mL)	1.55^a^	1.82^a^	0.96^b^	1.41^ab^	0.20	0.008*	0.040*	0.577
T4 (ng/mL)	46.79^ab^	51.70^a^	39.86^b^	43.94^ab^	3.47	0.017*	0.104	0.869
Insulin (μIU/mL)	6.35^ab^	7.86^a^	5.00^b^	6.16^ab^	0.69	0.014*	0.025*	0.731
Cortisol (ng/mL)	3.87^ab^	1.57^a^	7.35^b^	3.89^ab^	1.66	0.038*	0.039*	0.631

^1^Values are means and standard error of the means (*n* = 5).

^*ab*^Means in the same row with different superscripts differ (*P* < 0.05).

HDL-C, high-density lipoprotein cholesterol; LDL-C, low-density lipoprotein cholesterol; NEFA, none-esterified fatty acid; AST, aspartate aminotransferase; ALT, alanine aminotransferase; CON, control; RES, resveratrol; LPS, lipopolysaccharide; RES + LPS, dietary resveratrol treatment followed by LPS challenge.

The * indicates statistically significant difference (*P* < 0.05).

According to Table 4, the plasma hormone concentrations of T3, T4, and insulin was significantly decreased and plasma cortisol concentrations was significantly increased in yellow-feathered broilers (*P* < 0.05). In contrast, dietary supplementation with 400 mg/kg RES significantly reversed the decrease in plasma T3 and insulin concentrations and the increase in cortisol concentrations induced by LPS challenge (*P* < 0.05).

### Antioxidant capacity

The results of antioxidant capacity of plasma and jejunal mucosa are shown in Table 5. LPS challenge caused a significant decrease in plasma GSH-Px, SOD, CAT, and T-AOC activities and a significant increase in plasma MDA concentrations in yellow-feathered broilers (*P* < 0.05). Dietary supplementation with 400 mg/kg RES significantly the increase in plasma CAT and T-AOC activities caused by LPS (*P* < 0.05). Moreover, a significant increase in SOD and T-AOC activities was found in the jejunal mucosa of yellow-feathered broilers by LPS challenge (*P* < 0.05). Dietary supplementation with 400 mg/kg RES significantly increased jejunal SOD and T-AOC activities (*P* < 0.05). However, no significant LPS × RES interaction for these variables was observed. And there were no significant changes in plasma SOD activity as well as the CAT and MDA activities in jejunal mucosa among different treatments (*P* > 0.05).

**TABLE 5:** Effect of dietary resveratrol supplementation on antioxidant capacity in yellow-feathered broilers with lipopolysaccharide^1^.

Item	Treatment				SEM	*P*-value		
	CON	RES	LPS	RES + LPS		LPS	RES	LPS × RES
**Plasma**								
GSH-Px (U/mL)	872.65^a^	896.98^a^	713.50^b^	836.60^ab^	69.24	0.039*	0.152	0.328
SOD (U/mg)	175.96^a^	179.11^a^	140.71^b^	174.47^a^	12.73	0.042*	0.057	0.109
CAT (U/mL)	3.37^b^	4.23^a^	2.57^b^	3.15^b^	0.40	0.004*	0.021*	0.632
T-AOC (mmol/L)	0.58^b^	0.72^a^	0.43^c^	0.56^b^	0.05	<0.001*	0.001*	0.732
MDA (nmol/mL)	4.46^ab^	4.01^b^	5.18^a^	4.74^ab^	0.44	0.048*	0.189	0.979
**Jejunal mucosa**								
GSH-Px (U/mg prot)	197.79	209.22	181.74	193.13	18.11	0.228	0.386	0.999
SOD (U/mg prot)	29.57^ab^	33.84^a^	25.20^b^	30.64^ab^	3.25	0.054	0.022*	0.858
CAT (U/mg prot)	1.10	1.12	0.91	0.98	0.12	0.065	0.586	0.757
T-AOC (mmol/mg prot)	58.25^ab^	69.32^a^	46.89^b^	59.96^ab^	6.54	0.040*	0.019*	0.832
MDA (nmol/mg prot)	0.70	0.62	0.78	0.72	0.062	0.076	0.154	0.892

^1^Values are means and standard error of the means, *n* = 5 per treatment.

^*ab*^Means in the same row with different superscripts differ (*P* < 0.05).

CON, control; RES, resveratrol; LPS, lipopolysaccharide; RES + LPS, dietary resveratrol treatment followed by LPS challenge; GSH-Px, glutathione peroxidase; SOD, superoxide dismutase; CAT, catalase; T-AOC, total antioxidant capacity; MDA, malondialdehyde.

The * indicates statistically significant difference (*P* < 0.05).

### Intestinal morphology

**FIGURE 1:**
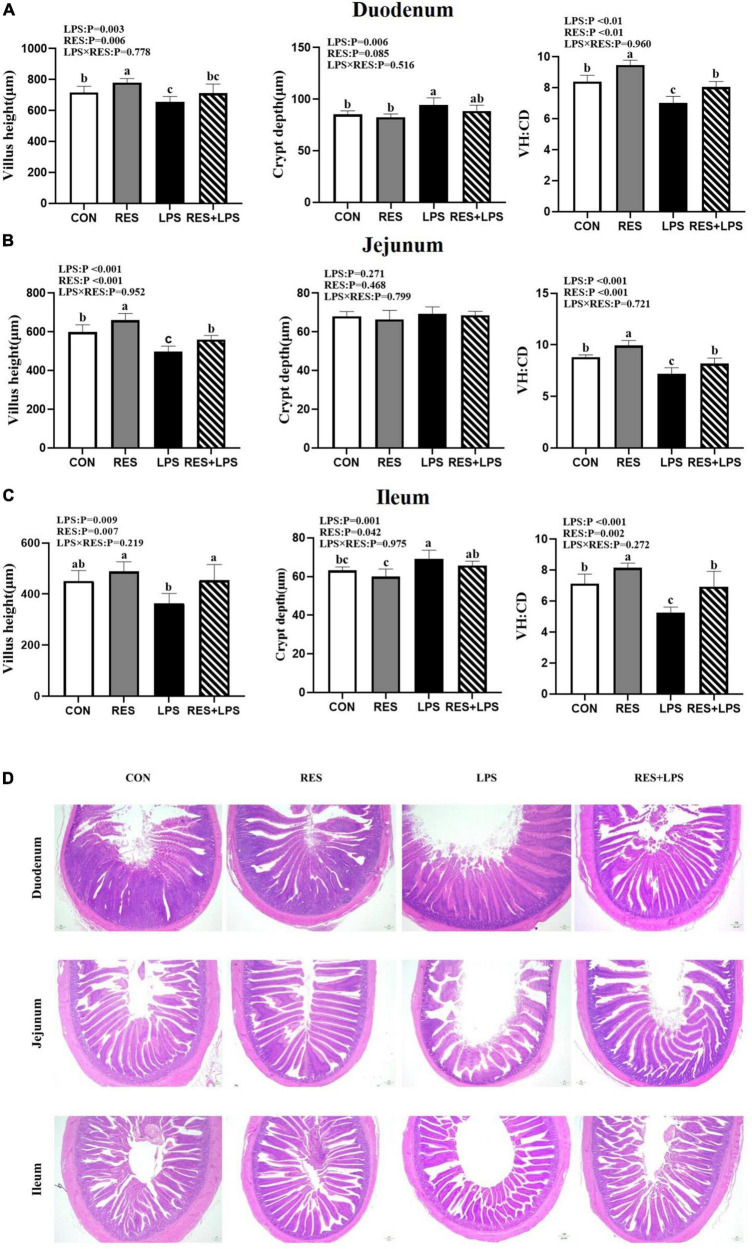
Effect of dietary resveratrol supplementation on intestinal morphology in yellow-feathered broilers with lipopolysaccharide. **(A)** Duodenum. **(B)** Jejunum. **(C)** Ileum. **(D)** The represent images of HE staining. Original magnification 40×, scale bar 20 μm. Values are means and standard error of the means, *n* = 5 per treatment. *^abc^* Means in the columns with different superscripts differ (*P* < 0.05). CON, control; RES, resveratrol; LPS, lipopolysaccharide; RES + LPS, dietary resveratrol treatment followed by LPS challenge.

The analysis of intestinal morphology (Figure 1) showed that LPS challenge resulted in intestinal damages in the duodenum, jejunum, and ileum of yellow-feathered broilers, as evidenced by a significant shortening of villus height and a highly significant decrease in villus height to crypt depth ratio of duodenum, jejunum, and ileum (*P* < 0.01) and an increase in crypt depth of duodenum and ileum (*P* < 0.05). However, the addition of RES at 400 mg/kg significantly alleviated the reduction in the ratio of villus height to crypt depth in the duodenum, jejunum and ileum and prevented the shortening of villus height in jejunum and ileum caused by LPS (*P* < 0.05), and significantly reduced the crypt depth in the ileum in yellow-feathered broilers (*P* < 0.05).

### The mRNA expression and secretion of cytokines in the jejunal mucosa

**FIGURE 2:**
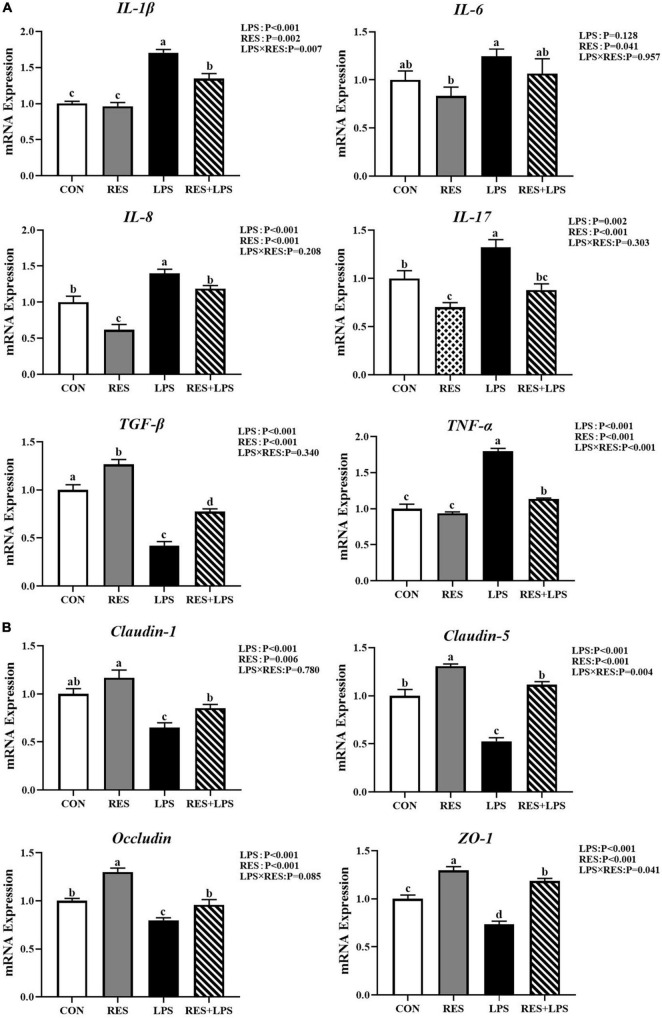
Effect of dietary resveratrol supplementation on intestinal gene expression in yellow-feathered broilers with lipopolysaccharide. **(A)** The mRNA expression of intestinal cytokines. **(B)** The mRNA expression of tight junctions. Values are means and standard error of the means, *n* = 5 per treatment. *^abc^* Means in the columns with different superscripts differ (*P* < 0.05). CON, control; RES, resveratrol; LPS, lipopolysaccharide; RES + LPS, dietary resveratrol treatment followed by LPS challenge.

The real-time PCR analysis (Figure 2A) showed that LPS challenge upregulated (*P* < 0.01) the mRNA expression levels of *IL-1*β, *IL-8*, *IL-17*, and *TNF-*α and downregulated (*P* < 0.01) the mRNA expression levels of *TGF-*β in the jejunum mucosa of yellow-feathered broilers. However, the addition of RES significantly alleviated the changes in the above inflammatory factors caused by LPS (*P* < 0.01). Specially, dietary supplementation with RES significantly decreased the mRNA expression levels of *IL-6*, *IL-8*, and *IL-17* in the jejunal mucosa of yellow-feathered broilers (*P* < 0.05).

The Elisa assays (Table 6) also showed that LPS challenge caused a significant increase in TNF-α level and a significant decrease in TGF-β level in the jejunal mucosa of yellow-feathered broilers (*P* < 0.05). In contrast, the addition of RES at 400 mg/kg significantly alleviated the significant elevation of jejunal TNF-α, IL-6, and IL-1β concentrations compared to LPS group (*P* < 0.05). However, there is no significant difference in IL-17 level at jejunal mucosa and no significant LPS × RES interaction for these variables observed in present study (*P* > 0.05).

**TABLE 6:** Effect of dietary resveratrol supplementation on cytokine secretion in jejunal mucosa of yellow-feathered broilers with lipopolysaccharide^1^.

Item	Treatment				SEM	*P*-value		
	CON	RES	LPS	RES + LPS		LPS	RES	LPS × RES
TNF-α (pg/mL)	1.71^b^	1.22^b^	2.52^a^	1.71^b^	0.30	0.007*	0.007*	0.479
IL-6 (pg/mL)	4.86^b^	4.18^b^	6.76^a^	4.70^b^	0.87	0.066	0.041*	0.276
IL-1β (pg/mL)	2.11^ab^	1.72^b^	3.22^a^	1.82^b^	0.57	0.157	0.043*	0.227
IL-17 (pg/mL)	6.66	5.23	7.94	6.21	1.15	0.186	0.71	0.853
TGF-β (ng/mL)	5.85^a^	6.86^a^	2.79^b^	5.56^ab^	1.21	0.034*	0.059	0.331

^1^Values are means and standard error of the means, *n* = 5 per treatment.

^*ab*^Means in the same row with different superscripts differ (*P* < 0.05).

CON, control; RES, resveratrol; LPS, lipopolysaccharide; RES + LPS, dietary resveratrol treatment followed by LPS challenge; TNF-α, tumor necrosis factor-α; IL-6, interleukin-6; IL-1β, interleukin-1β; IL-17, interleukin-17; TGF-β, transforming growth factor-β.

The * indicates statistically significant difference (*P* < 0.05).

### The mRNA expression of intestinal tight junctions

As shown in Figure 2B, LPS challenge significantly decreased (*P* < 0.01) the mRNA expression levels of *claudin-1*, *claudin-5*, *occludin*, and *ZO-1* in jejunal mucosa. Furthermore, dietary supplementation with RES at 400 mg/kg significantly increased the mRNA expression levels of *claudin-5*, *occludin*, and *ZO-1* in jejunal mucosa of yellow-feathered broilers (*P* < 0.01).

### Gut microbiota composition and diversity

**FIGURE 3:**
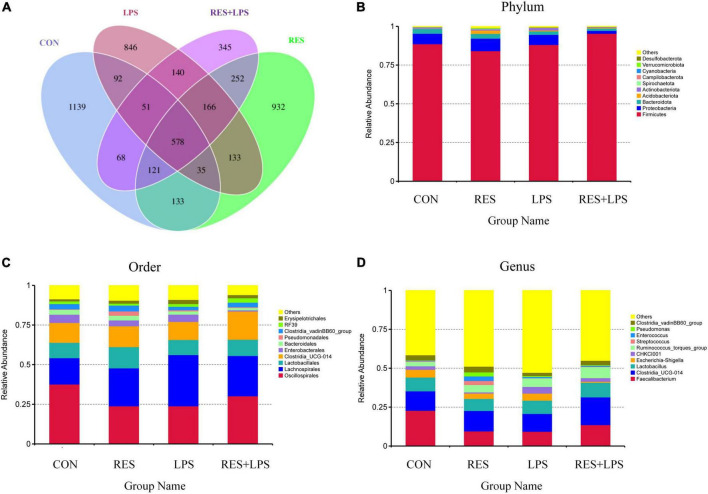
The Venn diagram and relative abundances of bacteria at the phylum, order, and genus levels. **(A)** Venn diagram. **(B)** Top 10 bacteria at phylum level. **(C)** Top 10 bacteria at order level. **(D)** Top 10 bacteria at genus order. CON, control; RES, resveratrol; LPS, lipopolysaccharide; RES + LPS, dietary resveratrol treatment followed by LPS challenge.

There were 1139, 846, 932, and 345 unique OTUs in the CON group, LPS group, RES group, and RES + LPS group, respectively. A total of 578 common OTUs were found among four treatment groups (Figure 3A). At the phylum level (Figure 3B), the top 10 phyla bacteria were Firmicutes, Proteobacteria, Bacteroidota, Acidobacteriota, Spirochaetota, Campylobacterota, Cyanobacteria, Verrucomicrobiota, Desulfobacterota, and others. At the order level (Figure 3C), the top 10 bacteria were *Oscillospirales*, *Lachnospirales*, *Lactobacillales*, *Clostridia_UCG-014*, *Enterobacterales*, *Bacteroidales*, *Pseudomonadales*, *Clostridia_vadinBB60_group*, *RF39*, *Erysipelotrichales*, and others. At the genus level (Figure 3D), the top 10 genera bacteria were *Faecalibacterium*, *Clostridia_UCG-014*, *Lactobacillus*, *Escherichia-Shigella*, *CHKC1001*, *Ruminococcus_torques_group*, *Streptococcus*, *Enterococcus*, *PseuDomonas*, *Clostridia_vadinBB60_group*, and others.

**FIGURE 4:**
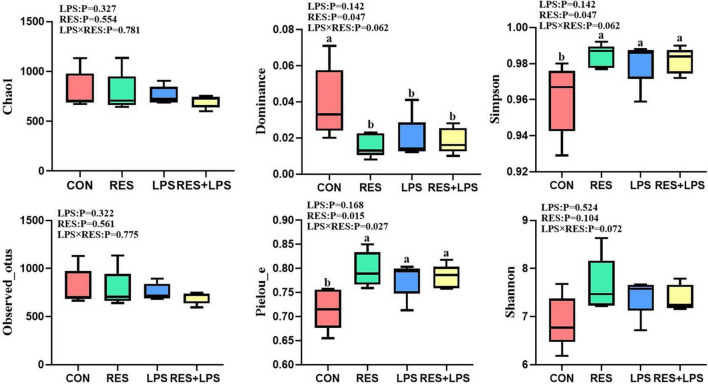
The effect of dietary resveratrol supplementation on the alpha diversity of cecal microbiota in yellow-feathered broilers. *^ab^* Means in the columns with different superscripts differ (*P* < 0.05). CON, control; RES, resveratrol; LPS, lipopolysaccharide; RES + LPS, dietary resveratrol treatment followed by LPS challenge.

As shown in Figure 4, the LPS challenge did not affect the alpha diversity of cecal microbiota in yellow-feathered broilers, but the RES treatments significantly decreased the dominance (*P* = 0.047) of cecal microbiota, and significantly increased the Pieiou-e (*P* = 0.015) and Simpson index (*P* = 0.047). There was significant interaction effect of LPS × RES in Pieiou-e of cecal microbiota of yellow-feathered broilers (*P* = 0.027).

**FIGURE 5:**
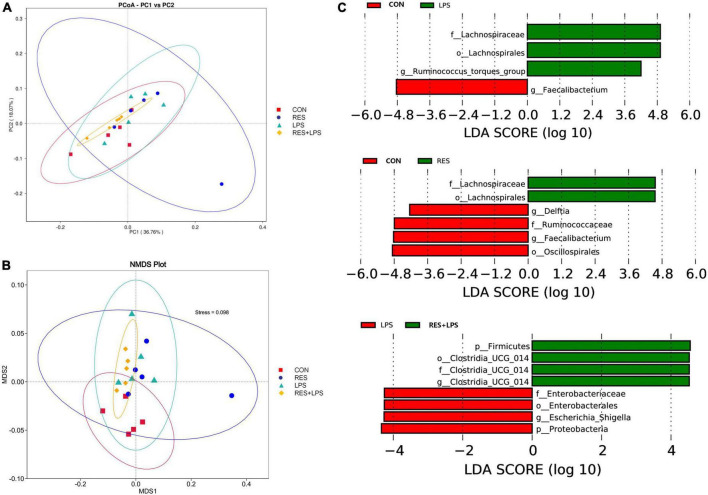
The effect of dietary resveratrol supplementation on the beta-diversity of cecal microbiota in yellow-feathered broilers. **(A)** The principal coordinate analysis (PCoA) plot; **(B)** Non-metric multidimensional scaling (NMDS) plot. **(C)** The LEfSe analysis (LDA score > 4). CON, control; RES, resveratrol; LPS, lipopolysaccharide; RES + LPS, dietary resveratrol treatment followed by LPS challenge.

The PCoA analysis (Figure 5A) and NMDS analysis (Figure5B) showed that the distances between CON group and LPS groups or between Control and RES groups were significantly distributed separately. The LEfSe analysis (Figure 5C) also indicated that three bacteria including *Lachnospiraceae* (Family), *Lachnospirales* (order), and *Ruminococcus_torques_group* (genus) were enriched in LPS when comparing to the CON group which enriched *Faecalibacterium* (genus). In contrast to control, LPS group enriched *Lachnospiraceae* (family) and *Lachnospirales* (order), while the CON group enriched *Deltia* (genus), *Ruminococcaceae* (family), *Faecalibacterium* (genus), and *Oscillospirales* (order). However, the RES + LPS group enriched four bacteria including Firmicutes (phylum), and *Clostridia_UCG-014* (order, family, and genus), while the LPS challenge enriched the *Enterobacteriaceae* (family), *Enterobacterales* (order), *Escherichia_Shigella* (genus), and Proteobacteria (phylum).

**FIGURE 6:**
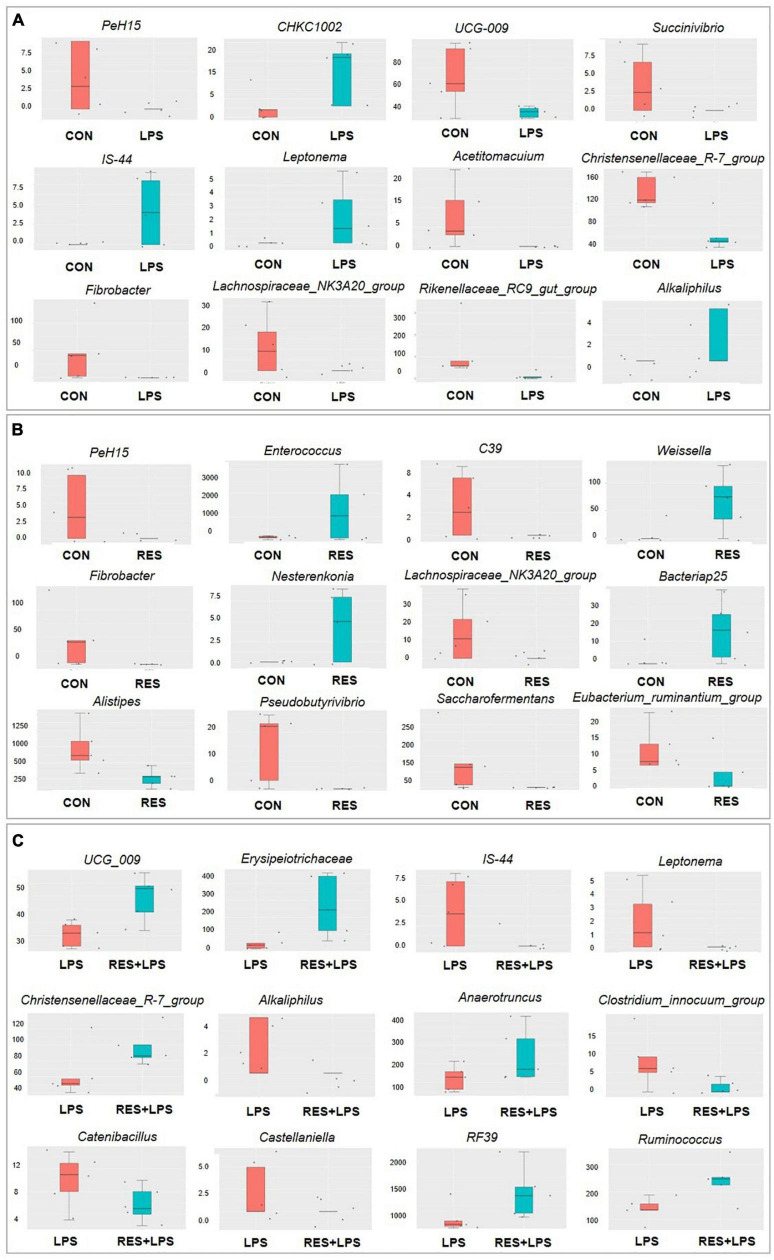
The Metastat analysis of cecal microbiota between treatments of cecal microbiota changes in yellow-feathered broilers. **(A)** Control group vs. LPS group. **(B)** Control group vs. RES group. **(C)** LPS group vs. RES + LPS group. CON, control; RES, resveratrol; LPS, lipopolysaccharide; RES + LPS, dietary resveratrol treatment followed by LPS challenge.

The Metastat analysis (Figure 6) showed that compared to the CON group, the relative abundances of *PeH15*, *UCG-009*, *Succinivibrio*, *Acetitomacuium*, *Christensenellaceae_R-7_group*, *Fibrobacter*, *Lachnospiraceae_NK3A20_group*, and *Rikenellaceae_RC9_gut_group* were significantly decreased while those of *CHKC1002, IS-44*, *Leptonema*, and *Alkaliphilus* were significantly increased in LPS group. Compared to the CON group, the relative abundances of *PeH15*, *C39*, *Fibrobacter*, *Lachnospiraceae_NK3A20_group*, *Alistipes*, *Pseudobutyrivibrio*, *Saccarofermentans*, and *Eubacterium_ruminantium_group* were significantly decreased while those of *Enterococcus*, *Weissella*, *Nesterenkonia*, and *Bacteriap25* were significantly increased in RES group. However, compared to the LPS group, the relative abundances of *IS-44*, *Leptonema*, *Alkaliphilus*, *Clostridium_innocuum_group*, *Catenibacillus*, and *Castellaniella* were significantly decreased while those of *UCG_009*, *Erysipelotrichaceae*, *Christensenellaceae_R-7_group*, *Anaerotruncus*, *RF39*, and *Ruminococcus* were significantly increased in RES + LPS group.

**FIGURE 7:**
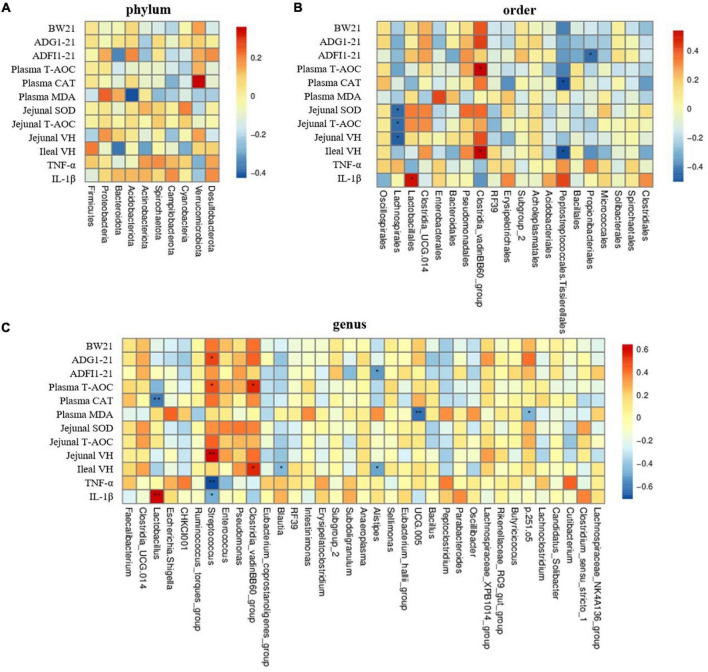
The Spearman correlation analysis of gut microbial composition at phylum, order, and genus levels with growth performance, antioxidant capacity, and intestinal health in yellow-feathered broilers. Spearman correlation coefficients of BW at 21 days, ADG and ADFI during 1–21 days, plasma and jenual antioxidant capacity, villus height at jejunum and ileum, and cytokine concentrations with representative cecal microbiota at phylum **(A)**, order **(B)**, and genus **(C)** level, are represented by color ranging from red (positive correlation) to blue (negative correlation), respectively. * and ^**^ indicates statistically significant difference (*P* < 0.05) and (*P* < 0.01), respectively. BW, body weight; ADG, average daily gain; ADFI, average daily feed intake; T-AOC, total antioxidant capacity; CAT, catalase; SOD, superoxide dismutase; MDA, malondialdehyde; VH, villus height; TNF-α, tumor necrosis factor-α; IL-1β, interleukin-1β.

The Spearman correlation analysis showed that there was no significant correlation between top 10 phyla bacteria with the representative significant phenotypes of the yellow-feathered broilers challenged with LPS (Figure 7A) (*P* > 0.05). However, at the order level, the relative abundance of *Lachnospirales* was negatively associated with jejunal SOD and T-AOC activities as well the villus height in the jejunum (Figure 7B) (*P* < 0.05). Moreover, the *Clostridia_vadinBB60_group* at both order and genus level was positively correlated with the plasma T-AOC activity and ileal villus height (Figures 7B,C) (*P* < 0.05). In addition, the intestinal IL-1β concentration was positively correlated with the relative abundance of *Lactobacillus* at both order (*P* < 0.05) and genus levels (*P* < 0.01) (Figures 7B,C), whereas the plasma CAT activity was negatively correlated with the *Lactobacillus* abundance at genus level (Figure 7C) (*P* < 0.01). Furthermore, the relative abundance of *Streptococcus* at the genus level was positively correlated with ADG at 1–21 days (*P* < 0.05), plasma T-AOC activity (*P* < 0.05) and jenunal villus height (*P* < 0.01), but was negatively correlated with intestinal TNF-α (*P* < 0.01) and IL-1β concentrations (Figure 7C) (*P* < 0.05). Additionally, the genera *Blautia* abundance was negatively associated with ileal villus height while plasma MDA concentration was negatively correlated with that of *UCG_005* and bacteria *p.251.05* (Figure 7C) (*P* < 0.05). Notably, there was a negative correlation between the relative abundance of genera *Alistipes* and ADFI at 1–21 days and ileal villus height in yellow-feathered broilers (Figure 7C) (*P* < 0.05).

## Discussion

Resveratrol is a natural polyphenolic compound with strong antioxidant and anti-inflammatory activities, which have been widely used for the prevention and treatments of many human diseases and disorders (Wang et al., 2016; Mayangsari and Suzuki, 2018). Increasing evidence also suggested that RES can effectively improve intestinal barrier function (Wang et al., 2022), alleviate the oxidative stress and intestinal inflammation for the enhancement of gut health and performance in piglets (Cao et al., 2019; Qiu et al., 2021), ducks (Yang C. et al., 2021), rats (Wang et al., 2022), and mice (Hao et al., 2022) under different experimental models. It has been well demonstrated that LPS could be used to mimick pathogen infection in broilers for studying immune inflammatory responses (Zhang et al., 2020, 2021; Erinle et al., 2022a). However, studies on the effects of RES on the growth performance, antioxidant capacity, intestinal health and gut microbiota of yellow-feathered broilers at early stage under LPS challenge conditions remained limited. Moreover, whether the protective of RES on broiler performance and health involved with the alterations of gut microbiota remained largely unknown.

Therefore, we investigated the effects of RES on the growth performance, antioxidant capacity and intestinal barrier function and gut microbiota in yellow-feathered broilers challenged with LPS. As expected, the present results showed that LPS significantly impaired the growth performance of yellow-feathered broilers by reducing ADG and ADFI and increasing the F/G during days 16–21. Consistently, previous studies have clearly demonstrated that immune stress induced by LPS excitation severely affected ADG and ADFI in broiler chickens; Yang S. et al., 2021; Zhang et al., 2021 (Kamboh et al., 2016; Li X. et al., 2021). Moreover, the induction of immune stress by LPS injection after 14 days significantly affected the growth performance in weaned piglets (Li Y. S. et al., 2021). Similarly, treatment with LPS for 3–10 times significantly reduced the ADG of rats (Zhong et al., 2018). However, we found that dietary supplementation with RES significantly improved the final BW, as well as the ADG and ADFI during days 16–21 and days 1–21, and significantly reduced the F/G during days 16–21 of yellow-feathered broilers, which indicated that dietary RES supplementation was able to mitigate the LPS-induced decline in growth performance of yellow-feathered broilers at early stage. Our results was consistent with the previous report on the protective effect of RES on the growth performance of broilers under heat stress (He et al., 2019b; Wang et al., 2021) or when receiving conventional vaccinations (Zhang et al., 2014). Additionally, these above results may strengthen the rationale for applying RES to broiler production to improve the growth performance of broilers under LPS challenge as observed in current study.

Plasma biochemical biomarkers are generally used to reflect the physiological and health status of animals. The concentrations of plasma AST and ALT indirectly indicate the health status of the liver and their abnormal activities imply potential hepatic tissue damage (Nyblom, 2004; Zhang et al., 2017a). The present results showed that the injection of LPS could increase the plasma AST level in yellow-feathered broilers. Our results were in accordance with previous study demonstrating an increase in plasma AST activity in response to stress in broilers injected with Salmonella-derived LPS (Erinle et al., 2022a). Previous study also showed that the activity of AST in yellow-feathered broilers was increased by heat stress (He et al., 2019b). Besides, triglyceride mainly participates in body fat and energy metabolism, and infection and inflammation can be leading to multiple alterations in lipid and lipoprotein metabolism (Khovidhunkit et al., 2004). Moreover, HDL-C and LDL-C possess a core structure composed of total cholesterol and triglyceride that associates with the hydrophobic portion of LPS (Feingold and Grunfeld, 2011). The present results showed that LPS challenge resulted in significant increases of plasma creatine kinase, HDL-C and triglyceride concentrations in yellow-feathered broilers, but dietary RES supplementation had no significant mitigating effect on the elevated plasma triglyceride levels, which is consistent with the previous study (Mohebodini et al., 2019). However, the current study showed that RES had a significant alleviating effect on the elevated creatine kinase-MB and creatinine concentrations induced by LPS stress. This is consistent with previous findings that mice pre-treated with RES almost completely counteracted the deleterious effects of LPS-induced creatinine elevation in the liver and kidney (Sebai et al., 2010).

Stressors often cause dynamic changes in the hormones in the hypothalamic-pituitary-adrenal (HPA) axis (Khilji et al., 2018). The activation of the HPA axis in broilers under heat stress conditions leads to elevated plasma glucocorticoid corticosterone (Quinteiro-Filho et al., 2012). Besides, thyroid hormones (T3 and T4) are major metabolic hormones of the body to regulate liver, skeletal muscles, and kidney energy metabolism, as well as maintenance of physiological body temperature (Decuypere et al., 2005). The current results showed that LPS significantly reduced the plasma concentrations of T3, T4 and insulin and significantly increased cortisol levels in yellow-feathered broilers. Our results were in consistent with previous study that heat stress increased the cortisol levels and decreased the T3 and T4 levels in broilers (He et al., 2019b). However, our study shows that dietary supplementation with RES could alleviate the increase of cortisol concentration and the decrease of T3 and insulin caused by LPS stimulation, which might partially explain the improved growth performance of broilers after RES supplementation. Similarly, previous study has shown that supplementation with RES can reduce serum cortisol, T3 and T4, which is beneficial in reducing stress in broilers (Zhang et al., 2017b). This suggested that dietary supplementation with RES might positively regulated the abnormal levels of plasma hormones under LPS challenge to support the enhancements in growth performance.

The broilers are prone to oxidative stress when invaded by pathogenic microorganisms or stimulated by the environment which lead to the imbalance of antioxidant system. In the present study, the plasma GSH-Px, SOD, CAT, and T-AOC activities were significantly reduced and MDA concentration was significantly increased by LPS challenge in yellow-feathered broilers, which is consistent with previous studies that significantly increased MDA levels and reduced GSH-Px and SOD activities in broilers under LPS stimulation (Li X. et al., 2021; Xing et al., 2021). However, RES has a strong antioxidant capacity to capture and scavenge free radicals, which helps to alleviate oxidative stress-induced intestinal damage. Previous study has confirmed that RES can promote the expression of antioxidant genes, regulate the expression of inflammatory cytokines, and can effectively inhibit the apoptosis of piglet intestinal epithelial cells and alleviate the oxidative stress caused by deoxynivalenol (Yang et al., 2019). In our study, dietary addition of RES alleviated the decrease in plasma CAT and T-AOC activities caused by LPS, while increasing the activities of SOD and T-AOC in jejunal mucosa and decrease plasma MDA concentration, which suggested the potential of RES in alleviating the LPS-induced oxidative damages in yellow-feathered broilers. These results might be partially attributed to the hepatoprotective effects and antioxidant capacity of RES (Yang C. et al., 2021). Similarly, dietary RES supplementation has been demonstrated to effectively alleviate the decrease of SOD and CAT activities induced by heat stress with reducing the concentration of MDA at the same time (Liu et al., 2014). These results confirm that RES could effectively exert its antioxidant function to ensure the intestinal health and growth of animals (Kim et al., 2011).

The gastrointestinal tract is not only the main site to digest and absorb nutrients, but also represents the biggest immune organ and endocrine organ in the body (Cannon et al., 2011). Villus height, crypt depth, and their ratio are often used as indicators of gut integrity and maintenance of normal intestinal function (Erinle and Adewole, 2022). Indeed, the increase in the villus height is closely related to enhanced digestive ability, thus promoting the growth and development of animals (Erinle et al., 2022b). In this study, LPS significantly decreased the villus height and the ratio of villus height to crypt depth in the duodenum, jejunum and ileum of broilers. We found that the addition of RES could increase the villus height, and alleviate the intestinal morphological damages induced by LPS, thus improving the intestinal mucosa integrity in yellow-feathered broilers. These results are consistent with previous studies on the protective effect of RES on intestinal morphology and structure (Liu et al., 2016; Zhuang et al., 2021; Hong et al., 2022). Moreover, the intestine mucosal barrier represents the first line of the host defence against invading enteric pathogens and toxins, which plays key roles in nutrient digestion and absorption (Turner, 2009). LPS stimulation can cause intestinal barrier damage, oxidation and immune stress, and then affect the intestinal development and growth performance of livestock and poultry (Gu et al., 2022). Tight junctions (TJs) are protein complexes in intestinal epithelial cells that can regulate intestinal epithelial permeability and maintain the intestinal barrier integrity (Suzuki, 2020). The most commonly recognized TJs included both the intracellular proteins zona occludens (*ZO-1*, *ZO-2*, and *ZO-3*) and transmembrane proteins (*claudins* and *occludin*) (Suzuki, 2013). In our study, LPS caused a decrease in the relative mRNA expression of *ZO-1*, *claudin-1*, and *claudin-5* in jejunal mucosa of yellow-feathered broilers. The previous results showed that LPS-induced duck ileitis accompanied with the destruction of intestinal structure and permeability alteration, severe oxidative stress, mitochondrial dysfunction, and inflammatory response (Wang et al., 2021; Yang H. et al., 2021). Dietary RES supplementation has been demonstrated to effectively alleviated LPS-induced intestinal dysfunction and the increase of intestinal permeability by linearly increasing mRNA levels of several tight junction protein genes (*claudin-1*, *occludin-1*, and *ZO-1*) as well as the protein expression of claudin-1 (Xun et al., 2021). In our study, the *claudin-1*, *occludin*, *ZO-1* mRNA expression was also significantly increased by RES treatment, which was consistent with a previous study in pigs (Chen et al., 2020). These above results suggested that RES might facilitate the maintenance of intestinal physical barrier function by increasing the expression of tight junction proteins.

The specificity and efficiency of the defense mechanism of intestinal immune response depend on the intestinal mucosa integrity (Ziegler et al., 2003). When the body is stimulated by microbial antigens such as LPS, it will stimulate macrophages, monocytes and endothelial cells to release a series of inflammatory mediators such as cytokines, oxygen free radicals and histamine to cause inflammatory reaction and reduce barrier function (Wu et al., 2015). The classical LPS cytokine cascade system includes IL-1β, IL-6, and TNF-α (Reisinger et al., 2021). In broilers, LPS could result in elevated inflammatory cytokines and reduced immune function, which lead to the damage to the gut integrity and thus causes intestinal immune dysfunction and impaired performance (Kamboh and Zhu, 2014; Wu et al., 2015). In this study, compared with the control group, LPS increased the concentration of TNF-α, IL-6, and IL-1β in jejunal mucosa of broilers, and decreased the concentration of TGF-β. Similarly, LPS significantly increased the mRNA expression of *IL-1*β, *IL-8*, *IL-17*, and *TNF-*α in jejunal mucosa. In addition, pro-inflammatory cytokines are effective stimulators of the HPA axis. It has been shown that excess of these cytokines may increase energy consumption and reduce food intake (Dec et al., 2020), which might account for the decline in growth performance of broilers by LPS observed in this experiment. However, we found that dietary RES inhibited the increase of the concentration of TNF-α, IL-6, and IL-1β, and increased the TGF-β concentration, as well as inhibiting the mRNA expression of *IL-1*β, *IL-8*, *IL-17*, and *TNF-*α in jenual mucosa. These results suggested that dietary RES supplementation might effectively reduce the intestinal inflammation to alleviate intestinal injury induced by LPS. Similarly, Qian et al. (2021) also showed that RES could attenuate the prostatic inflammation and reduce the expression of *IL-6*, *IL-8*, and *TNF-*α in rat with oestradiol-induced chronic prostatitis.

The maintenance of the dynamic balance of gut microbiota plays an important role in alleviating inflammation and improving animal intestinal health. The change of cecal microbial community is an important index of intestinal non-specific immune response (Jiang et al., 2021). Firmicutes, Bacteroidetes, Proteobacteria, Actinobacteria, and Cyanobacteria are the main phyla of broiler (Chen et al., 2020). Moreover, previous studies have shown that the addition of RES can regulate the balance of intestinal microflora (Sung et al., 2017; Cai et al., 2020). Consistently, our results showed that RES + LPS group increased the abundance of Firmicutes and decreased the abundance of the Proteobacteria compared to the LPS group. Similarly, previous study found that RES (200 mg per kg/day) attenuated the intestinal microflora dysbiosis in mice fed with a high-fat diet especially by increasing the ratio of Bacteroides to Firmicutes ratio, and enhancing the abundance of *Lactobacillus* (Qiao et al., 2014). Moreover, at the genus level, RES treatments significantly increased the relative abundances of *Erysipelotrichaceae* and *Ruminococcus* in the cecal digesta of yellow-feathered broilers. Previous study has demonstrated that the abundance of intestinal *Erysipelotrichaceae* are positively correlated with carbohydrate consumption (Cox et al., 2012), while the abundance of *Erysipelotrichaceae* and *Ruminococcus* has a positive association with short-chain fatty acids (SCFAs) levels (Kong et al., 2019; Li L. et al., 2020). Moreover, we found that the relative abundance of *Lachnospirales* negatively associated with jejunal SOD and T-AOC activities as well the villus height in the jejunum by Spearman correlation analysis. Importantly, *Lachnospiraceae* (family) and *Lachnospirales* (order) was found to be enriched in LPS-induced immune stress group, coupled with reduced antioxidant capacity and impaired intestinal morphology found in current study. Accordingly, a recent study also revealed close correlations between bacterial communities belonging to *Bacteroidales* and *Lachnospirales* orders and putative reactive aldehydes covariated with oxidative stress and leaky gut phenotype in mice (Mazenc et al., 2022). Furthermore, another study also showed that the abundance of butyrate-producing bacteria *Lachnospiraceae* was significantly reduced in rats with stress-induced visceral hypersensitivity (Zhang et al., 2019). However, due to the fact that SCFAs play an important role as an energy substrate to influence the host energy metabolism (Besten et al., 2013), it requires further investigations to clarify whether RES could regulate the SCFAs production and energy metabolism of broilers by modulating the gut microbiota community.

In addition, dietary supplementation with RES significantly reduced the abundance of genera *Alistipes* in the cecal digesta of yellow-feathered broilers when compared to the control group. *Alistipes* is highly associated with dysbiosis and inflammation and has been identified as a potential opportunistic pathogen for many diseases, such as liver fibrosis, colorectal cancer, cardiovascular disease, and mood disorders (Rodríguez-Nogales et al., 2017; Kong et al., 2019). Here, the significant negative correlations were found between the relative abundance of cecal *Alistipes* and ADFI during days 1–21 and villus height in the ileum. So, the reduced abundances of *Alistipes* by dietary supplementation with RES might be associated with the restore of intestinal morphology and improvement of performance in yellow-feathered broilers. Our results was consistent with previous study in layer pullets demonstrating a significant negative correlation between growth parameters (including ADFI and feed conversion ratio) and Alistipes (Liu et al., 2021). Furthermore, the microbial alpha diversity analysis showed that RES treatments significantly decreased the dominance of cecal microbiota, but increased the Simpson index and Pieiou-e in yellow-feathered broilers, indicating RES impact on regulation of microbial diversity. This was in accordance with previous study that dietary RES altered microbial composition and restored microbial community diversity in dextran sulfate sodium-treated mice (Li F. et al., 2020). In summary, dietary supplementation with RES could cause dynamic changes in the bacterial community as well as microbial diversity in the cecal digesta in yellow-feathered broilers under LPS challenge at early stage, which might be associated with the enhancement of the broiler growth performance and antioxidant capacity, and restore intestinal health and immunity disturbed by LPS.

## Conclusion

Collectively, the present results suggest that dietary supplementation with 400 mg/kg of RES could improve the growth performance, antioxidant capacity and immunity, which might be closely linked with the alterations in the gut microbiota community composition in yellow-feathered broilers. The results of this study may provide scientific basis for the application of RES as a feed additive for protecting intestinal health and performance in yellow-feathered broilers. However, further investigations are necessary to elucidate the potential mechanism of gut microbiota as biomarker targets interacting with animal performance and wellbeing through nutritional intervention strategies.

## Data availability statement

The datasets presented in this study can be found in online repositories. The data can be found below: Submission ID: SUB11567250, NCBI BioProject number: PRJNA846164, available at: http://www.ncbi.nlm.nih.gov/bioproject/846164/.

## Ethics statement

This animal study was reviewed and approved by Animal Care and Use Committee of Foshan University.

## Author contributions

ZH, YL, TX, and XN performed the methodological research. ZH, YL, and TX data analysis and wrote the original manuscript. ZH, YL, TX, CZ, and HZ wrote, reviewed, and edited the manuscript. CZ and HZ contributed to conceptualization and obtaining the funding. All authors contributed to the article and approved the submitted version.
